# Metabolites and Metabolic Functional Changes—Potential Markers for Endothelial Cell Senescence

**DOI:** 10.3390/biom14111476

**Published:** 2024-11-20

**Authors:** Jingyuan Ya, Alison Whitby, Ulvi Bayraktutan

**Affiliations:** 1 Stroke, Academic Unit of Mental Health and Clinical Neurosciences, School of Medicine, Queens Medical Centre, University of Nottingham, Nottingham NG7 2UH, UK; jingyuan.ya@nottingham.ac.uk; 2 Children’s Brain Tumor Research Centre, School of Medicine, Biodiscovery Institute, University of Nottingham, Nottingham NG7 2RD, UK; 3 School of Medicine, Ankara Medipol University, Hacı Bayram Mah, Talatpaşa Blv No. 4, 06050 Altındağ, Türkiye

**Keywords:** endothelial cells, metabolomics, metabolites, aging, senescence, biomarkers, age-related disease

## Abstract

Accumulation of senescent endothelial cells (ECs) in vasculature represents a key step in the development of vascular aging and ensuing age-related diseases. Given that removal of senescent ECs may prevent disease and improve health and wellbeing, the discovery of novel biomarkers that effectively identify senescent cells is of particular importance. As crucial elements for biological pathways and reliable bioindicators of cellular processes, metabolites demand attention in this context. Using senescent human brain microvascular endothelial cells (HBMECs) displaying a secretory phenotype and significant morphological, nuclear, and enzymatic changes compared to their young counterparts, this study has shown that senescent HBMECs lose their endothelial characteristics as evidenced by the disappearance of CD31/PECAM-1 from interendothelial cell junctions. The metabolic profiling of young versus senescent HBMECs also indicates significant differences in glucose, glutamine, and fatty acid metabolism. The analysis of intracellular and secreted metabolites proposes L-proline, L-glutamate, NAD^+^, and taurine/hypotaurine pathway components as potential biomarkers. However, further studies are required to assess the value of these agents as potential biomarkers and therapeutic targets.

## 1. Introduction

Cerebrovascular diseases continue to be one the leading causes of mortality and disability-adjusted life years worldwide [1,2]. Aging represents the major risk factor for the development of cerebrovascular diseases and accounts for much of the higher mortality and morbidity rates associated with these conditions [3]. Hence, identification and specific targeting of key mechanisms or elements contributing to the pathogenesis of age-related vascular diseases (ARVDs) are of critical importance for the delay of disease onset and extension of healthy lifespan [4]. Recent evidence suggests vascular aging, accompanied by accumulation of senescent cells, as one such key mechanism [5].

Endothelial cells (ECs) cover the entire inner surface of all blood vessels and constitute one of the main cellular components of the vascular tree. They synthesize a large number of vasoactive agents in response to various chemical, humoral, and physical stimuli and thus help maintain vascular hemostasis at all times [6]. ECs acquire different morphologies and functional characteristics in different organs whereby they adapt to different microenvironments and perform highly specific functions [7,8]. For instance, in the brain, the microvascular ECs (BMECs) form tight junctional complexes between neighboring cells and help establish a specific barrier called the blood–brain barrier (BBB), which regulates the selective passage of compounds between the blood and the central nervous system [9]. When senescent, BMECs acquire new morphological and nuclear characteristics, lose their capacity to self-renew, fail to form tight junctions, gradually accumulate in vasculature, and consequently promote vascular aging [10,11]. However, senescent BMECs remain metabolically active and continue to release a wide range of inflammatory cytokines and chemokines, growth factors, and enzymes that collectively make up a senescence-associated secretory phenotype or SASP [12]. Once established, the SASP promotes senescence in adjacent cells to reinforce the senescent state.

Cellular metabolism comprises both anabolic and catabolic reactions, including those relating to redox hemostasis, energy production, and biomass synthesis [13]. An increasing number of evidence indicates that metabolic reprogramming during senescence is a necessity to channel substantial quantities of energy towards specific activities coupled to the senescent state, including the SASP and increased oxidative stress and endoplasmic reticulum stress [14,15]. Intriguingly, young ECs generate most of their energy from the anaerobic conversion of glucose to lactate despite the presence of copious amount of oxygen in the vasculature [16]. EC senescence is likely to affect this relatively high glycolytic rate. High levels of oxidative stress, accompanied by excessive availability of reactive oxygen species, in senescent cells may also compromise the glycolytic flux and divert glycolytic intermediates into various pathological pathways by triggering DNA damage and stimulating the activity of NAD^+^-consuming enzymes.

In view of the above facts, using the metabolomics technology, the current study specifically investigated how metabolic functions and metabolite profiles changed in BMECs with senescence. Our intention was to evaluate whether any of the key metabolites identified may serve as reliable biomarkers for vascular aging, a precursor for the onset and development of various ARVDs, in future studies.

## 2. Materials and Methods

### 2.1. Cell Culture

Human BMECs (HBMECs) were purchased from Neuromics (Minneapolis, MN, USA). The cells were cultured in a humidified atmosphere (75% N_2_, 20% O_2_, 5% CO_2_) at 37 °C in an endothelial cell medium (SC-1001, ScienCell Research Laboratories, Carlsbad, CA, USA).

Throughout the study, HBMECs at passage 7 or 8 were used as young or non-senescent cells. Replicative senescence was induced through the repetitive passaging of young HBMECs. Senescence was deemed to exist when over 70% of cultured cells stained positive for SA-β-gal and γ-H2AX. This normally occurred at passage ≥18.

### 2.2. SA-β-Galactosidase Activity

A total of 60,000 young or senescent HBMECs were plated in 12-well plates and cultured until they were about 70% confluent. The activity of S-β-galactosidase (SA-β-gal) was evaluated using a beta-galactosidase staining kit (ab102534, Abcam, Cambridge, UK). In brief, fixed cells were incubated overnight in the staining solution at 37 °C. Cells with blue dye under light microscope were considered senescent. To detect the changes in β-galactosidase activity across different study groups, cells were visualized and photographed by light microscopy (Leica DFC3000 G, Leica Microsystems, Wetzlar, Germany) using 20× magnification. Cells were counted manually in a minimum of four randomly chosen areas of each slide before calculating the percentage of SA-β-gal-positive cells for each slide. Each experiment was repeated at least three times using three different biological replicates.

### 2.3. Proliferation Assay

The proliferation capacity of young and senescent HBMECs was evaluated by the WST-1 (4- [3- (4-lodophenyl)-2- (4-nitrophenyl)-2 H-5-tetrazolio]-1.3-benzene disulfonate) Cell Proliferation Assay kit (Roche, Mannheim, Germany). The WST-1 assay is based on the cleavage of the tetrazolium salt WST-1 to formazan by mitochondrial dehydrogenases in viable cells. Higher absorbance units (AUs) indicate larger numbers of viable cells. In brief, 5 × 10^3^ passage 8 or passage 18 HBMECs were seeded in 96-well plates and cultured for 24 h. The culture media was subsequently replaced with 100 μL fresh media containing 10 μL of WST-1 assay reagent. The plates were incubated for 2 h at 37 °C prior to reading the absorbance (at 480 nm) by a FLUOstar Omega microplate reader (BMG Labtech Ltd., Aylesbury, UK).

### 2.4. Immunocytochemistry

Young and senescent HBMECs were seeded on glass coverslips and cultured to 80% confluence before fixing and permeabilizing in 4% paraformaldehyde and 0.1% Triton X-100, respectively. The cells were then blocked with 1% bovine serum albumin in PBST (0.1% Tween20 in PBS) at room temperature for 30 min before incubating with primary antibodies, namely, phosphor-Histone H2AX (Ser139, 9718, Cell Signaling Technology, Danvers, MA, USA) and CD31/PECAM-1 (3528, Cell Signaling Technology, Danvers, MA, USA), at 4 °C overnight. On the next day, the cells were washed and incubated with FITC-labeled or Texas Red-labeled secondary antibodies (ab6785, ab6719, Abcam, Cambridge, UK) for 1 h at room temperature in the dark. To visualize F-actin microfilaments, the cells were incubated with the DyLight 594 Phalloidin (12877, Cell Signaling Technology, Danvers, MA, USA) for 15 min after the blocking step.

After washing, the cells were incubated with DAPI (4,6-diamidino-2-phenylindole) for 3 min for nuclei staining. The coverslips were mounted on glass slides using a mounting medium (Vector Laboratories, Peterborough, UK) and visualized by a fluorescence microscope (Leica DFC3000 G, Leica Microsystems, Wetzlar, Germany). Nuclei with red-colored foci were regarded as γH2AX-positive cells. By counting all DAPI-stained nuclei, total cell numbers were determined. Cells were counted manually in at least four randomly selected areas of each slide before calculating the percentage of γH2AX-positive cells for each slide. Each experiment was repeated at least three times using three different biological replicates.

### 2.5. Tubulogenesis Assay

The angiogenic capacity of the HBMECs was evaluated by the extent of tubule network formed on Matrigel (Corning, New York, NY, USA). Matrigel was thawed overnight at 4 °C and pipetted into 96-well plates (50 μL per well) using pre-chilled tips before letting it solidify for 1 h at 37 °C. HBMECs (8 × 10^3^ cell/150 μL culture medium) were then seeded on the Matrigel and incubated at 37 °C for 4 h. The formation of the tubular structures was photographed by Digipad connected to a light microscope (Leica DFC3000 G, Leica Microsystems, Wetzlar, Germany). The total number of tubes formed and the total length of tubular segments were processed and quantified using Angiogenesis Analyzer plugin ImageJ software (version 1.52k, NIH, Bethesda, MD, USA)

### 2.6. Wound Healing Assay

Young and senescent HBMECs were seeded in 6-well plates (1.5 × 10^5^ cells/well) and grown to 90% confluence. The wound was created by scratching the cell monolayer across with a p1000 micropipette tip in one swift motion. Debris were washed away with warm PBS. Pictures of the wound were captured immediately (0 h) and after 24 h of incubation in complete media using the Digipad connected to a light microscope (Leica DFC3000 G, Leica Microsystems, Wetzlar, Germany). Wound closure was quantified as the percentage of difference in the scratch area between 0 and 24 h using the ImageJ software (version 1.53k, NIH, Bethesda, MD, USA).

### 2.7. Protein Extraction and Quantification

Following exposure to various experimental conditions, the cells were washed with ice cold PBS (x2) before scraping with 1x RIPA lysis buffer containing 1 mM sodium orthovanadate and 10 μL/mL protease inhibitor cocktail. The supernatant was separated from the cell lysate via centrifugation at 14,000× *g* (4 °C) for 15 min. Protein concentrations were determined using the BCA protein assay kit (23227, Thermofisher, Waltham, MA, USA) according to the manufacturer’s instructions.

### 2.8. Western Blotting

Total protein samples (20–80 μg) were mixed with 4% lithium dodecyl sulfate sample buffer (MPSB, Sigma, Burlington, MA, USA) in a ratio of 3:1 and heated for 5 min at 75 °C to open the tertiary/quaternary structures. After electrophoresis on SDS polyacrylamide gels, the protein samples were transferred to a PVDF membrane on ice. The membrane was blocked for 1 h with 5% BSA at room temperature and incubated overnight with the primary antibodies at 4 °C. In this study, specific antibodies raised against p16 (ab51243, Abcam, Cambridge, UK) and β-actin (ab8226, Abcam, Cambridge, UK) were employed as primary antibodies. The membranes were then washed and incubated with IRDye-labeled (800 CW/680 CW) secondary antibodies (926-32211, 926-68070, Licor, Li-cor Biotechnology, Lincoln, NE, USA). The protein bands were detected using the Odyssey Fc Imager, and their intensities were quantified by Image Studio software (5.0, Li-cor Biotechnology, Lincoln, NE, USA).

### 2.9. Telomere Length Measurement

Genomic DNA was isolated from young and senescent HBMECs through the DNeasy Blood & Tissue Kit (69504, Qiagen, Germantown, MD, USA). Telomere length was measured using the Absolute Human Telomere Length Quantification qPCR Assay Kit (ScienCell Research Laboratories, Carlsbad, CA, USA). The average length of telomeres per chromosome end was worked out according to manufacturer’s instructions using the formula based on Cq values acquired by Stratagene MX3000P Quantitative RT-PCR System (Agilent, Santa Clara, CA, USA).

### 2.10. Analysis of the Secretory Cytokines

Culture media was collected from young and senescent HBMECs as described in previous studies [17,18]. Briefly, confluent cells were cultured in media lacking both the serum and growth factors for 24 h. The supernatants were then collected and condensed 8 times by centrifugation at 4000× *g* for 20 min using a centrifugal filter (Merck, Taufkirchen, Germany). To screen and analyze the level of cytokines, a Proteome Profiler Human Cytokine Array Kit (ARY005B, R&D Systems, Minneapolis, MN, USA) was used. The chemiluminescent signal on the membrane was captured using the Odyssey Fc system and quantified by ImageJ software (5.0, National Institutes of Health, Bethesda, MD, USA).

### 2.11. Liquid Chromatography Mass Spectrometry (LC-MS) Metabolomics Analysis

From a T25 flask of confluent cells, a sample of media (1 mL) was taken and flash frozen to later extract the metabolic footprint. The remaining media was removed from the flask, and the cells washed twice with PBS (5 mL, 37 °C) [19]. Methanol (500 μL, −80 °C, LC-MS grade) was added to the T25 flasks (6 repetitions for each group). The flasks were rocked and swirled gently on ice for 3 min, and then, the cells were scraped into Eppendorf tubes. The cell lysate was centrifuged at 13,000× *g* for 30 min at 4 °C, and the supernatant (500 µL) was filtered using a 5 kD molecular weight cut-off PES filter column (pre-washed with water) at 12,000× *g* for 45 min at 4 °C. Filtrate was collected for LC-MS. A flask containing media but no cells was processed in the same way as a reagent blank. Protein concentration was determined from further replicate T25 flasks using the BCA assay kit.

Defrosted spent media samples were centrifuged at 10,000× *g* for 5 min at 4 °C, and then, supernatant (250 μL) was mixed with methanol (750 μL, −20 °C, LC-MS grade) followed by incubation at −20 °C for 20 min and a second vortex (15 s) [19]. Finally, sample was centrifuged at 17,000× *g* for 10 min at 4 °C, and supernatant was collected for LC-MS.

The analytical run followed the untargeted metabolomics method published previously, and data were processed as described previously with a mass list created for this analytical run and data normalized to the amount of protein per sample (determined from a parallel culture) [20]. Protein amount differed between non-senescent and senescent samples, with 266.5 µg and 237.4 µg, respectively. Identification levels 1–4 were assigned to metabolites according to the accuracy of annotation as described previously.

### 2.12. Statistical Analysis

Data are presented as mean value ± standard error of the mean (SEM) from a minimum three independent experiments. Level of changes across different study groups were determined using unpaired *t*-test. *p <* 0.05 was considered as significant. The quality of the liquid chromatography mass spectrometry data was described by the tight clustering of the QC data from repeated injections on the principal component analysis (PCA) score plot. PCA and orthogonal partial least squares discriminant analysis (OPLS-DA) were undertaken as multivariate analyses. *T*-test with Benjamini–Hochberg false discovery rate (FDR) correction was used as univariate analysis. The metabolites responsible for the difference between groups were determined using both multivariate [variable important for the projection (VIP) ≥ 1 in OPLS-DA] and univariate (adjusted *p*-value *<* 0.05) statistics.

## 3. Results

### 3.1. Late-Passage Endothelial Cells Exhibit Morphological Change and Express Markers of Cellular Senescence

**Figure 1:**
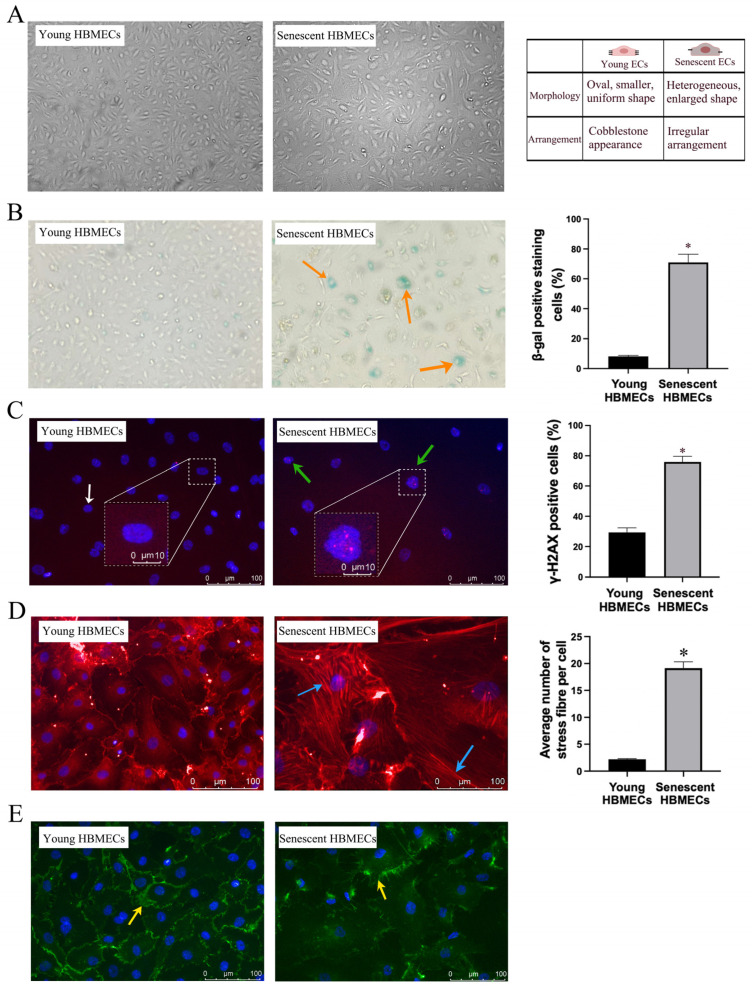
Senescence promotes morphological and functional changes in human brain microvascular endothelial cells (HBMECs) as evidenced by enlarged, heterogenous morphology (**A**) and increases in β-gal activity (**B**, orange arrow) and DNA damage accumulation (**C**, green arrow). White arrow shows normal nuclear staining with DAPI. Senescent HBMECs develop greater numbers of actin stress fibers (**D**, blue arrow) and lose their endothelial cell characteristics as evidenced by a decrease in CD31 expression (**E**, yellow arrow). * *p <* 0.05 compared to young HBMECs.

Compared to early-passage HBMECs, late-passage cells (passage 18) were significantly larger and flat. Furthermore, late-passage cells displayed more heterogeneous morphology and possessed significantly higher SA-β-gal activity and γ-H2AX expression, a sensitive marker for DNA double-strand breaks (Figure 1A–C). Further analysis of cellular structural organization through actin microfilament staining confirmed that senescent cells possessed a large number of actin stress fibers traversing the cells, explaining the flattened and enlarged morphology observed in these cells (Figure 1D). With an increasing passage number, the expression of endothelial marker CD31 diminished in HBMECs (Figure 1E). Taken together, these findings proved that most cells at passage 18 were senescent.

**Figure 2:**
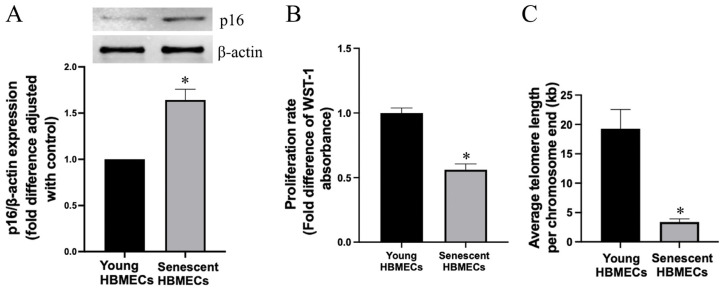
Senescent human brain microvascular endothelial cells (HBMECs) express significantly higher levels of p16 (**A**) and a lower proliferation rate (**B**) and a lesser telomere length (**C**) compared to young HBMECs. * *p <* 0.05 compared to young HBMECs. Original images of (**A**) can be found in Supplementary Materials.

In addition to morphological changes, senescent cells were also shown to express substantially higher levels of p16, a cyclin-dependent kinase inhibitor that is critical for regulating the cell cycle (Figure 2A). Indeed, measurement of cell proliferation as an index of cell cycle progression confirmed the decline in proliferative capacity of senescent HBMECs (Figure 2B) that also had significantly shorter telomeres (Figure 2C), stemming from the progressive attrition of telomeres with repetitive passaging [21].

### 3.2. Senescent Endothelial Cells Lose Their Angiogenic Potential

**Figure 3:**
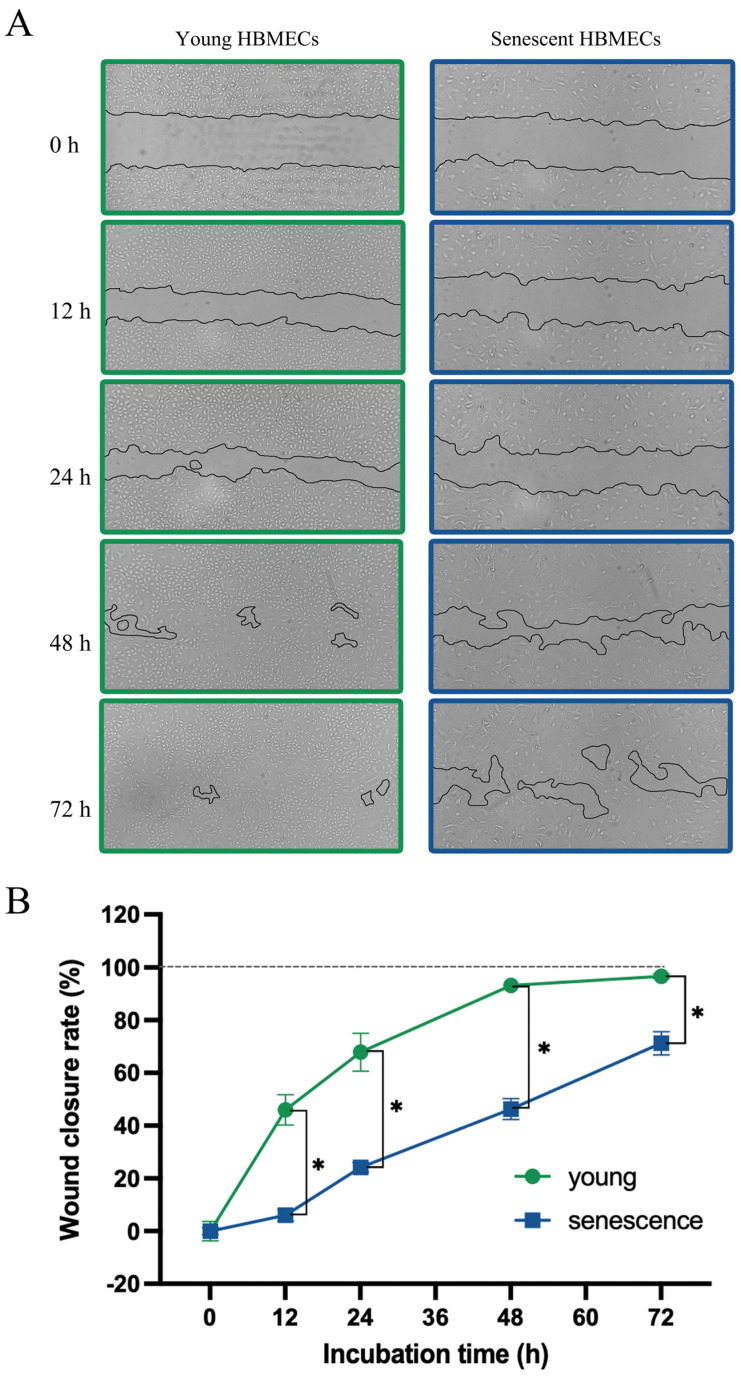
Senescent human brain microvascular endothelial cells display significantly reduced migratory capacity (**A**) as evidenced by extended time required to repair the wound inflicted on HBMEC monolayer (**B**). * *p <* 0.05 compared to young HBMECs.

Angiogenesis is a process in which new blood vessels form from the existing ones through synchronized migration, proliferation, and differentiation of ECs [22]. Similar to its impact on proliferative capacity, senescence also impaired the migratory capacity of HBMECs as evidenced by a markedly slower rate of wound repair (Figure 3A,B).

**Figure 4:**
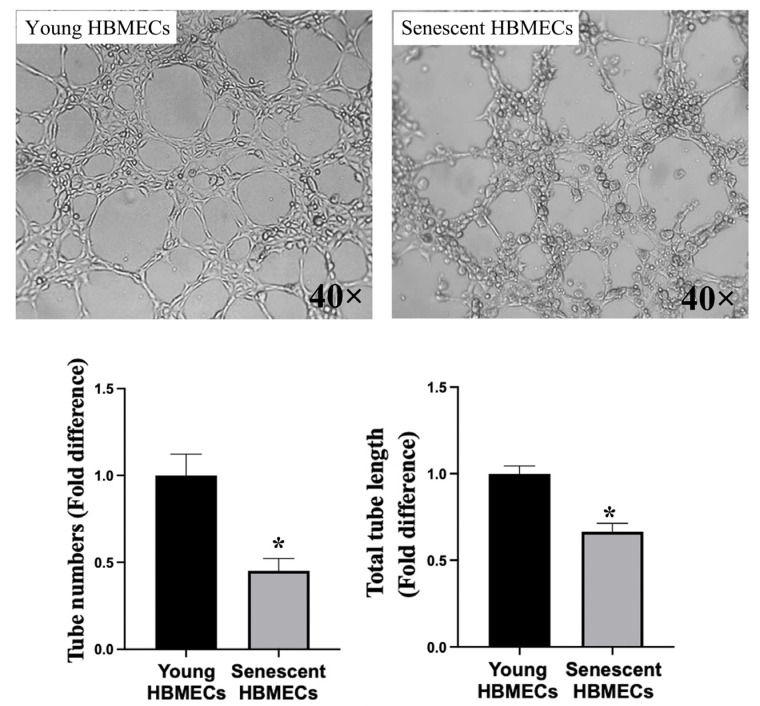
Senescent human brain microvascular endothelial cells (HBMECs) display impaired angiogenic capacity as ascertained by their ability to form fewer, shorter, and irregular tubules on Matrigel compared to their young counterparts. * *p* < 0.05 compared to young HBMECs.

Direct scrutiny of the angiogenic capacity of young versus senescent HBMECs via tubulogenesis assay, assessing both the total number and length of the capillary-like structures formed on Matrigel, showed that senescent cells developed fewer and shorter tubules on Matrigel (Figure 4).

### 3.3. Production of Inflammatory Cytokines Is Elevated in Senescent HBMECs

**Figure 5:**
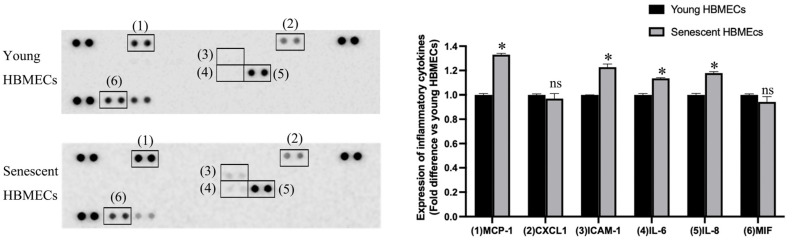
Compared to young human brain microvascular endothelial cells (HBMECs), senescent cells release greater quantities of inflammatory cytokines, MCP-1, ICAM-1, IL-6, and IL-8. * *p <* 0.05 compared to young HBMECs. ns = not significant.

Senescence in HBMECs evoked an inflammatory microenvironment, characterized by increased release of inflammatory cytokines, monocyte chemoattractant protein-1 (MCP-1), intercellular adhesion molecule-1 (ICAM-1), interleukin-6 (IL-6), and IL-8 (Figure 5).

### 3.4. Cellular Senescence Results in Changes in Metabolite Abundance in Endothelial Cells

**Figure 6:**
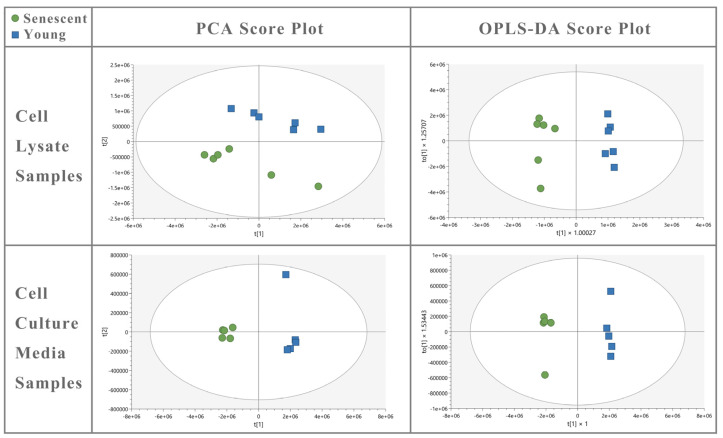
Untargeted metabolite fingerprinting (cell lysate) and footprinting (spent media) in senescent = 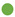 and young 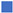 HBMEC lysate and culture media samples. OPLS-DA of cell lysate (R^2^Y = 0.980; Q^2^ = 0.969) and media (R^2^Y = 0.995; Q^2^ = 0.985) shows a good fit of data and the predictive ability of the model without overfitting.

The cell lysate and media samples from senescent and young ECs revealed good separation in PCA and OPLS-DA score plots, substantiating the quality of the liquid chromatography mass spectrometry data (Figure 6).

### 3.5. Intracellular Metabolite Profile of HBMECs Changes with Senescence

Metabolites that exhibit differential abundance between senescent and young ECs are summarized in Table 1. In the intracellular metabolites analysis, two metabolites were determined to have significantly increased abundance with senescent ECs compared to the young cells, namely, ethanolamine phosphate and choline phosphate, used in transforming diacylglycerol lipids into phosphatidylethanolamine and phosphatidylcholine lipids, transforming them from storage lipids to membrane lipids with polar or charged head groups. Meanwhile, four metabolites revealed significantly decreased abundance, including L-aspartate, a precursor to several amino acids, L-glutamate, a crucial energy source under glucose deficiency, L-proline, an essential component of collagen, and sn-glycero-3-phosphocholine, a precursor to choline biosynthesis. In addition, 22 metabolites displayed significantly lower abundance in senescent HBMEC lysates only by *t*-test, including the molecules indicative of energetic metabolism especially in the mitochondrion (AMP, NAD^+^, malate, O-acetylcarnitine, 2-oxoglutarate, pantothenate, β-alanine), metabolites in the taurine and hypotaurine metabolism pathway (taurine, hypotaurine), and other metabolic products that participate in the catabolism and synthesis reactions in the body. N-Acetyl-L-aspartate abundance elevated significantly in senescent HBMECs by *t*-test. sn-Glycero-3-phosphocholine, sn-glycero-3-phosphoethanolamine, and sn-glycerol 3-phosphate were decreased in abundance in senescent cells, the former two being the first metabolites of phosphatidylcholine and phosphatidylethanolamine lipids catabolism, and the latter the second metabolite in phosphatidylcholine catabolism.

### 3.6. The Profile of Metabolite Secretome of HBMECs Changes with Senescence

In the cell culture media, lactate, a byproduct of glucose metabolism as well as a stimulator of mitochondrial electron transport chain (ETC) activity [23], showed significant decline in senescent cells’ media. Senescent HBMECs display significantly lower release of another four metabolites by *t*-test only, including guanine, hypoxanthine (both purine bases), 2′-deoxycytidine, and O-succinyl-homoserine, and increased production of L-thyronine, 3- or 4-methyl-2-oxopentanoate, and 3-methyl-2-oxobutanoate, with the latter two being the first intermediates of branched-chain amino acid (BCAA) degradation (Table 2).

## 4. Discussion

To date, several elements, relying mostly on differences between young and senescent cell morphology, telomere length, and enzymatic activity, have been proposed as biomarkers to detect senescence in vitro and in vivo [24]. Since abundance and/or activity of most of these markers are modulated by distinct physio-pathological stimuli and differ from one cell type to another, it is of critical importance to discover novel markers that can reliably detect senescent cells in both experimental and clinical settings irrespective of differences in cellular origin and status of health [25]. The present study explored whether metabolic function or metabolite profile changes between young and senescent HBMECs. If yes, whether the magnitude of changes warrant the consideration of certain elements as potential biomarkers and necessitate their analyses at the organismal level in future studies. Given the close association between BMEC senescence and vascular aging, a precursor to many neurovascular degenerative diseases, this is a seminal issue.

To ensure that HBMECs employed in the outlined studies were truly senescent, a panel of markers were used to identify these cells. In accordance with the previous studies [26,27], HBMECs, deemed to be senescent in the present study, also displayed enlarged and heterogenous morphology, developed actin stress fibers, expressed significantly greater levels of p16 protein, a cyclin-dependent kinase inhibitor, SA-β-gal activity, and γ-H2AX foci, a sensitive marker of DNA damage. Furthermore, the senescent cells demonstrated a markedly shorter telomere length and reduced proliferative and migratory capacities [11,24]. Concurrent declines in the plasma membrane staining of CD31/PECAM-1, a marker for endothelial cells, revealed that senescent HBMECs lost their endothelial characteristics.

A wide range of distinct stimuli, including oxidative stress, hyperglycemia, fatty acid oxidation, hypoxia, and disturbances in shear stress, has been implicated in EC dysfunction. This paper illustrates that cellular senescence may also contribute to this process through a mechanism that requires the exaggerated release of MCP-1, IL-6, IL-8, and soluble ICAM-1 by senescent cells. Indeed, out of 36 inflammatory cytokines and chemokines targeted by multianalyte assays, only the levels of these particular cytokines appeared to be constantly upregulated in the secretome of HBMECs subjected to replicative senescence. ECs have been shown to orchestrate excessive cytokine release in other physio-pathological conditions such as during viral infections [28,29,30,31,32,33,34]. It is likely that the inflammatory microenvironment resulting from the accumulation of senescent BMECs help promote age-related arterial stiffness, tight junction dissolution, and neurovascular disease formation [10,35] and needs to be counterbalanced to maintain vascular equilibrium.

The balance among anabolism, catabolism, and waste removal is a crucial step for the maintenance of cellular homeostasis and relevant organ function [36]. Significant differences observed in the availability of certain metabolites, such as ethanolamine phosphate and choline phosphate, between young and senescent HBMECs pinpoint alterations in the metabolic function at the cellular level. Deteriorated angiogenesis and migratory capacity in senescent HBMECs accompanied by a disruption in tubule formation and decelerated wound healing confirm these functional differences. Even though glycolysis and oxidative phosphorylation (OXPHOS) make up the main energy sources of ECs, growing evidence indicates that, during the angiogenesis process, the ATP production of ECs is highly dependent on aerobic glycolysis rather than OXPHOS [37]. The decreased release of lactate into the media with senescent HBMECs suggest the suppression of aerobic glycolysis and explain the diminished angiogenesis capacity. The significantly increased production of choline phosphate and ethanolamine phosphate in senescent cells along with the decreased production of glycerophospholipid breakdown products suggest enhanced phosphatidylcholine (PC) and phosphatidylethanolamine (PE) synthesis. PE functions as the essential component in multiple physiological processes in eukaryotic cells, including PC synthesis, promoting cell membrane fusion, oxidative phosphorylation, and mitochondrial biogenesis [38]. It has been reported that membrane lipids including PC and PE are significantly upregulated in senescent conditions, which is accordance with our data, suggesting senescence-associated alteration in EC plasma membrane composition and dynamics. Firstly, most of the PC, PE, and sphingomyelin lipids were increased in abundance in exosomes from senescent primary human lung fibroblasts induced by senescence inducers [39]. Secondly, eight lyso-PCs and lyso-PEs were biomarkers of replicative senescence in mesenchymal stromal cells (MSCs) [40]. Proline is an important component of collagen formation and mitochondrial respiratory and protein folding stabilization [41,42]. Declined L-proline abundance was observed in the lysate sample of senescent HBMECs. A supplement of proline has been reported to delay senescence of mesenchymal stem cells [42]. Senescent HBMECs produced a significantly lower level of L-glutamate, which has been reported to trigger the release of nitric oxide, a potent vasodilator and an antioxidant, in brain ECs [43]. Senescent cells have impaired antioxidant capacity and increased reactive oxygen species generation [44],which may be associated with the decreased production of metabolites with antioxidant function reported in this study, including L-pyroglutamic acid, N-acetyl-L-methionine, and taurine. 2-oxoglutarate (α-ketoglutarate) was increased in abundance in MSCs from late passage [45], whereas 2-oxoglutarate and its reduced form, 2-hydroxyglutarate, were decreased in abundance in late passage in our HBMECs. A reduction in the amount of NAD^+^ was seen in HBMECs upon senescence, and similarly, a lower amount of nicotinamide riboside and nicotinamide ribonucleotide was observed in MSCs [45]. Lowered NAD^+^ levels and a lower cytosolic NAD^+^/NADH ratio lead to senescence and can occur by many mechanisms including inhibition of nicotinamide phosphoribosyltransferase, mitochondrial dysfunction, consumption by CD38, and inhibition of the malate–aspartate shuttle, potentially seen as lowered levels of malate in our experiment [46]. NAD^+^ levels decrease with increasing age in myriad tissues [46]. The metabolites in lower abundance in senescent MSCs were especially associated with the glycerophospholipid, TCA cycle, and taurine and hypotaurine pathways [45]. All of these metabolites were also noticeably lower in abundance in our HBMECs. Whilst the metabolites associated with the valine, leucine, and isoleucine (BCAA) biosynthesis pathway were in higher abundance in senescent MSCs, two metabolites of BCAA degradation (also named in the BCAA biosynthesis pathway) were in higher abundance in media from our HBMECs, corroborating our work. BCAAs were associated with elevated cystatin C, a SASP and proinflammatory marker; also, N-acetylserine was positively correlated with cystatin C, and cystatin C was elevated in the serum of rapid agers [47].

Tight junctional formation between neighboring BMECs is necessary to establish a tight BBB and regulate the selective passage of compounds to and from the brain. Loss of BBB integrity during the aging process may somewhat contribute to the development or exacerbation of neurodegenerative disorders. The disappearance of CD31/PECAM-1 from interendothelial cell junctions and the augmentation of the PC and PE synthesis pathway, in this study, suggest altered cell membrane unity and loss of cell–cell contact. The metabolic alterations in BMECs are coincident with the loss of tight junctions and therefore may be markers of BMEC senescence and the problems associated with tight junctional loss.

### Limitations and Future Work

There are some limitations in this study. These include the use of a single EC line. To examine whether there is a BMEC-specific metabolic profile of senescence, future experiments may also employ ECs obtained from other organs. Due to differences in the sizes of young and senescent BMECs, metabolomics data in the present study were normalized to the protein concentrations. As cell numbers may also influence metabolomics data, in future studies, an equal number of cells may be processed at the time of extraction.

Given the nature of replicative senescence, induced by repetitive passaging, other mechanisms are unlikely to substantially contribute to the differences observed in metabolites profile. Furthermore, to attribute these metabolic changes to senescence per se, time-course experiments designed to demonstrate gradual change(s) in the levels of these metabolites, through passages, may be undertaken. Application of other models of senescence, such as those induced by transfection of p16 or by exposure to oxidative stress or inflammatory cytokines, may add further weight to the correlation between the acquisition of senescent state and outlined metabolic changes. Finally, performance of additional quantitative experiments, such as ELISA or Western blotting, would have been good to further corroborate the differences detected in the levels of certain inflammatory cytokines, notably, MCP-1, ICAM-1, and IL-6, by a proteome profiler human cytokine array kit.

## 5. Conclusions

This study demonstrates that senescence is intimately associated with changes in EC phenotype and function, including proliferation, migration, and angiogenesis. The changes observed in certain intracellular and extracellular metabolites levels, notably, L-proline, L-glutamate, NAD^+^, and taurine/hypotaurine pathway components, could be targeted in future in samples from patients manifesting symptoms of cerebrovascular aging and age-matched healthy controls, to determine any applicability in vivo. Translational studies employing young and chronologically aged animals may also be of value to prove or dismiss the potential value of these elements as biomarkers.

## Figures and Tables

**Table 1:** Metabolites with differential abundance between young and senescent ECs in the cell lysate.

Annotation	Formula	ID Confidence Level *	Fold Change (Senescent/Young)	FDR Corrected *p*-Value	VIP Value
Hypotaurine	C_2_H_7_NO_2_S	3	0.49	8.45 × 10^−9^	0.38
N-Acetyl-L-glutamate	C_7_H_11_NO_5_	2	0.15	6.51 × 10^−7^	0.70
Taurine	C_2_H_7_NO_3_S	2	0.60	1.67 × 10^−6^	0.81
2-Aminoprop-2-enoate	C_3_H_5_NO_2_	4	0.52	6.53 × 10^−6^	0.18
L-Pyroglutamic acid	C_5_H_7_NO_3_	2	0.63	6.53 × 10^−6^	0.47
(Z)-2-Aminobutenoate	C^4^H_7_NO_3_	4	0.66	1.77 × 10^−5^	0.40
Ethanolamine phosphate	C_2_H_8_NO_4_P	2	7.29	2.63 × 10^−5^	1.43
UDP-N-acetyl-D-galactosamine	C_17_H_27_N_3_O_17_P_2_	4	0.57	3.99 × 10^−5^	0.36
N-Acetyl-L-methionine	C_7_H_13_NO_3_S	4	0.40	4.21 × 10^−5^	0.41
N-Methyl-L-glutamate	C_6_H_11_NO_4_	4	0.71	8.62 × 10^−5^	0.21
L-Aspartate	C_4_H_7_NO_4_	2	0.53	8.62 × 10^−5^	1.23
L-Glutamate	C_5_H_9_NO_4_	2	0.64	8.62 × 10^−5^	2.56
O-Acetyl-L-serine	C_5_H_9_NO_4_	2	0.62	9.36 × 10^−5^	0.43
NAD^+^	C_21_H_29_N_7_O_14_P_2_	2	0.65	9.78 × 10^−5^	0.25
O-Acetyl-L-homoserine	C_6_H_11_NO_4_	4	0.48	1.87 × 10^−5^	0.31
N-Acetyl-L-aspartate	C_6_H_9_NO_5_	4	1.82	3.56 × 10^−4^	0.57
sn-Glycero-3-phosphoethanolamine	C_5_H_14_NO_6_P	4	0.79	3.70 × 10^−4^	0.51
Malate	C_4_H_6_O_5_	2	0.59	3.70 × 10^−4^	0.99
β-Alanine	C_3_H_7_NO_2_	4	0.50	7.16 × 10^−4^	0.68
2-Oxoglutarate	C_5_H_6_O_5_	4	0.65	4.41 × 10^−3^	0.34
L-Proline	C_5_H_9_NO_2_	2	0.55	5.77 × 10^−3^	4.59
sn-Glycero-3-Phosphocholine	C_8_H_20_NO_6_P	2	0.82	8.36 × 10^−3^	2.93
Pantothenate	C_9_H_17_NO_5_	4	0.74	1.10 × 10^−2^	0.31
Palmitoyl ethanolamide	C_18_H_37_NO_2_	4	0.69	1.47 × 10^−2^	0.25
AMP	C_10_H_14_N_5_O_7_P	2	0.75	2.03 × 10^−2^	0.11
O-Acetylcarnitine	C_9_H_17_NO_4_	2	0.59	3.17 × 10^−2^	0.39
sn-Glycerol 3-phosphate	C_3_H_9_O_6_P	4	0.85	4.21 × 10^−2^	0.18
Choline phosphate	C_5_H_14_NO_4_P	2	1.24	4.69 × 10^−2^	1.46
(R)-2-Hydroxyglutarate	C_5_H_8_O_5_	2	0.79	4.80 × 10^−2^	0.19

* Level 1 is the highest. Levels 3 and 4 are putative identifications (IDs).

**Table 2:** Metabolites with differential abundance between young and senescent ECs in the spent media.

Annotation	Formula	ID Confidence Level *	Fold Change (Senescent/Young)	FDR Correct d *p*-Value	VIP Value
Guanine	C_5_H_5_N_5_O	4	0.52	3.94 × 10^−6^	0.20
Lactate	C_3_H_6_O_3_	2	0.56	2.64 × 10^−5^	5.72
Hypoxanthine	C_5_H_4_N_4_O	2	0.93	1.02 × 10^−4^	0.55
L-Thyronine	C_15_H_15_NO_4_	4	1.41	1.57 × 10^−4^	0.03
2′-Deoxycytidine	C_9_H_13_N_3_O_4_	3	0.35	2.73 × 10^−4^	0.15
3/4-Methyl-2-oxopentanoate	C_6_H_10_O_3_	4	1.44	1.90 × 10^−3^	0.69
3-Methyl-2-oxobutanoate	C_5_H_8_O_3_	4	1.31	3.43 × 10^−3^	0.31
O-Succinyl-homoserine	C_8_H_13_NO_6_	4	0.76	1.06 × 10^−2^	0.18

* Level 1 is the highest. Levels 3 and 4 are putative identifications (IDs).

## Data Availability

Data are available on reasonable request from the authors.

## Supplementary Materials

The following supporting information can be downloaded at: https://www.mdpi.com/article/10.3390/biom14111476/s1, Original Western Blots.
