# Supplementary figures and images for: Metabolites and Metabolic Functional Changes—Potential Markers for Endothelial Cell Senescence
Source: Biomolecules. 2024 Nov 20;14(11):1476. doi: 10.3390/biom14111476 (PMC11592342; doi:10.3390/biom14111476)

WB 1

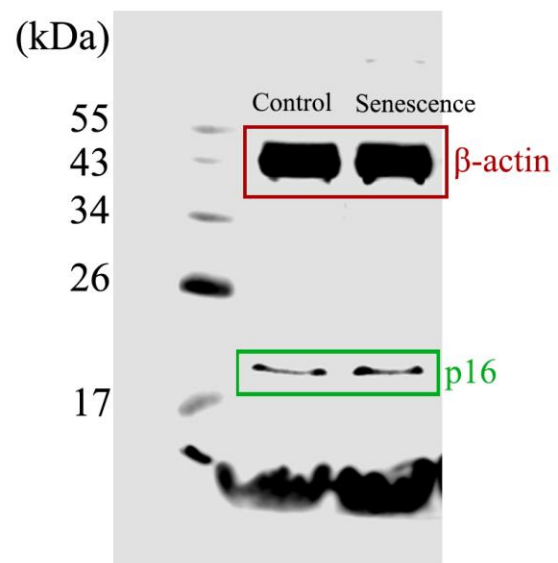

WB 2

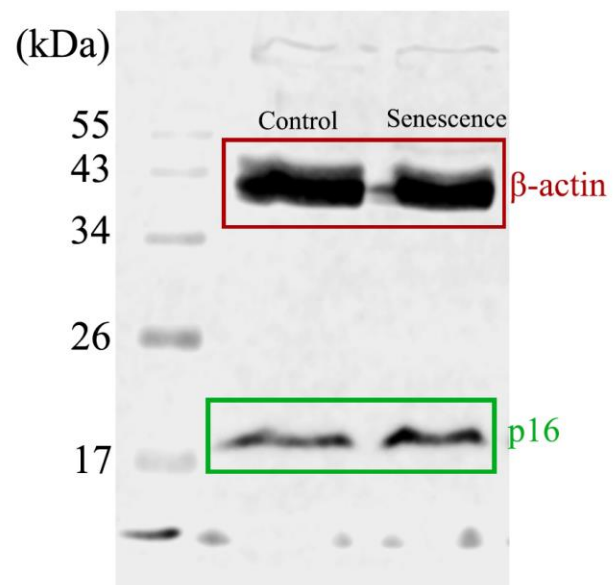

WB 3

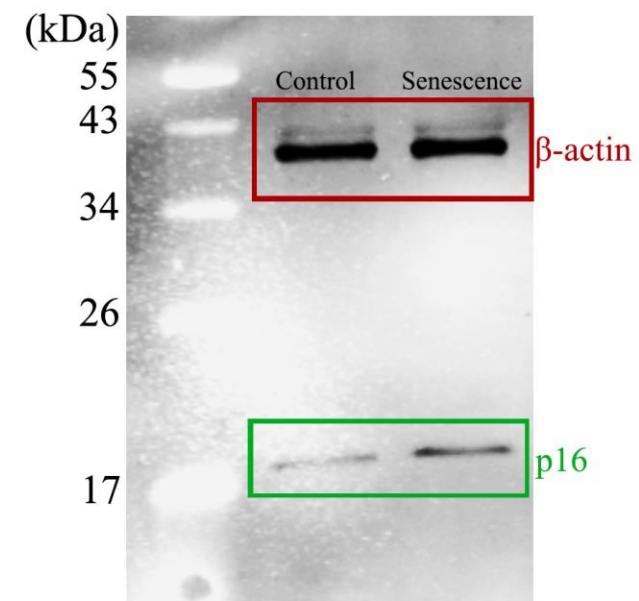

Supplement: Supplementary file 1 [file biomolecules-14-01476-s001.zip › biomolecules-3257460-supplementary.pdf]
